# Neuroprotective efficacy of ABCA1 and HDL in aging rats with cerebrovascular hypoperfusion

**DOI:** 10.1186/1750-1326-7-S1-S2

**Published:** 2012-02-07

**Authors:** Linhui Wang, Mingyuan Tian, Gang Yu, Hongchi Luo, Yu Li

**Affiliations:** 1 Department of Pathology, Chongqing Medical University, Chongqing 400016, China; 2 Institute of Neuroscience, Chongqing Medical University, Chongqing 400016, China; 3 Chongqing Key Laboratory of Neurobiology, Chongqing Medical University, Chongqing 400016, China; 4 Department of Neurology, the First Affiliated Hospital, Chongqing Medical University, Chongqing 400016, China

## Background

To establish an animal model of Vascular dementia(VD), which produced by permanent, bilateral occlusion of the common carotid arteries(2VO) in aging rats, and to study the expression of ABCA1 protein in hippocampal CA1 and the levels of serum HDL and TC in a vascular dementia model.

## Method

Brain hypoperfusion was induced by 2VO. Dynamic changes of ABCA1 in hippocampus were detected by immunohistochemistry. Serum levels of HDL and TC in different groups of rats were measured by automatic biochemical analyzer.

## Result

The levels of serum HDL and TC increased at 2 weeks post-surgery compared with sham-operated group (P<0.05). Then, the levels decreased to the lowest at 4 weeks accompanied by the prolonged ischemia compared with 2 weeks post-surgery (P<0.05). However, the expression of ABCA1 showed significant difference in the CA1 of hippocampus at 1 week after 2VO compared with sham-operated group (P<0.05). It slightly increased accompanied by recovery of cerebral blood flow (CBF) at 2 weeks compared with sham-operated group (P>0.05). The expression of ABCA1 reached to the peak at 3 weeks (P<0.01). At 4 weeks, the expression of ABCA1 began to decrease, it showed significant different compared with sham-operated (P<0.01).

## Conclusion

We demonstrated that ABCA1 and HDL underwent dynamic expression in hippocampus of rats’ brain after 2VO. It suggested that ABCA1 and HDL might play protective roles in vascular dementia. Our study gave further evidence for clarifying the underlying mechanism of Lipid metabolism in vascular dementia, but more data are needed to firmly confirm this protective effect.

